# SIRT3 promotes lipophagy and chaperon-mediated autophagy to protect hepatocytes against lipotoxicity

**DOI:** 10.1038/s41418-019-0356-z

**Published:** 2019-06-03

**Authors:** Tian Zhang, Jingxin Liu, Shengnan Shen, Qiang Tong, Xiaojun Ma, Ligen Lin

**Affiliations:** 1 State Key Laboratory of Quality Research in Chinese Medicine, Institute of Chinese Medical Sciences, University of Macau, Macau, China; 2 Children’s Nutrition Research Center, Department of Pediatrics, Baylor College of Medicine, Houston, TX USA; 3 Department of Endocrinology, The First Affiliated Hospital of Zhengzhou University, Zhengzhou, Henan China

**Keywords:** Nutrition disorders, Metabolic disorders

## Abstract

Lipophagy is a lysosomal lipolytic pathway that complements the actions of cytosolic neutral lipases. Chaperon-mediated autophagy (CMA) triggers lipid droplets (LDs) breakdown, to initiate lipolysis via either cytosolic lipases or macroautophagy. SIRT3, a mitochondrial NAD^+^-dependent deacetylase, regulates the acetylation status and activity of many substrates involving in energy metabolism. However, the role of SIRT3 in regulating lipophagy is controversial. The current study showed that SIRT3 expression was decreased and the macroautophagy flux was blocked in the primary hepatocytes from high-fat diet fed mice and P/O (palmitic acid and oleic acid mixture) treated AML12 mouse hepatocytes, compared with the corresponding controls. SIRT3 overexpression promoted macroautophagy in LDs from P/O-treated hepatocytes through activating AMP-activated protein kinase (AMPK) and unc-51-like kinase 1, to boost LDs digestion. Gain of SIRT3 expression stimulated the formation of lysosome-associated membrane protein 2A (LAMP-2A)-heat shock cognate 71 kDa protein (HSC70)-perilipin-2 (PLN2) complex, to promote CMA process and reduce the stability of LDs in hepatocytes. Moreover, SIRT3 reduced the expression of stearoyl-CoA desaturase 1, to suppress lipogenesis. In addition, SIRT3 overexpression promoted LDs dispersion on detyrosinated microtubules, and directly deacetylated long-chain acyl-CoA dehydrogenase to enhance mitochondrial energetics. Taken together, SIRT3 ameliorates lipotoxicity in hepatocytes, which might be a potential target for the treatment of nonalcoholic fatty liver disease.

## Introduction

Nonalcoholic fatty liver disease (NAFLD) is increasing in an alarming rate worldwide [1]. Ectopic lipid accumulation in hepatocytes and subsequent lipotoxicity play a crucial role in the onset and development of NAFLD [2]. Autophagy is an evolutionarily conserved recycling process to maintain intracellular energy homeostasis via a lysosome-dependent degradation. Impaired autophagy has been documented to associate with conditions that predispose to NAFLD in both experimental and clinical studies [3–5]. Increasing evidence has indicated that lipid droplets (LDs) are selectively recognized by the macroautophagic machinery and efficiently incorporated into autophagosomes to release free fatty acids (FFAs), a process termed lipophagy [5, 6]. Macroautophagy provides a potential mechanism for alleviating lipotoxicity in hepatocytes.

The perilipins (PLINs) are the most abundant LD coat proteins [7]. PLIN2 is the constitutive LDs protein in hepatocytes. PLIN2 abundance is closely associated with the level of intracellular lipid, and PLIN2 protein is highly expressed in the liver from fatty liver patients [8]. Recently, PLIN2 was reported to participate in the chaperon-mediated autophagy (CMA) [7]. CMA is initiated with the recognition of the cytosolic heat shock cognate protein (HSC70) complex by the CMA receptor lysosome-associated membrane protein 2A (LAMP-2A), and then the lysosome complex binds to PLIN2 to engulf LDs into lysosomes lumen for lysosomal degradation. In addition, degradation of PLIN2 by CMA is required for the initiation of lipolysis via either cytosolic lipases or macroautophagy, through AMP-activated protein kinase (AMPK)-dependent phosphorylation of PLIN2 [9].

SIRT3 is an NAD^+^-dependent deacetylase that regulates energy metabolism and oxidative stress [10]. The murine SIRT3 gene expresses both long-form mitochondrial and short-form cytosolic SIRT3 [11–13]. Emerging evidence has indicated that SIRT3 plays an important role in regulating lipid homeostasis and improving NAFLD [14–16]. However, the underlying mechanisms of the protective effect of SIRT3 against NAFLD remain elusive. Hererin, we investigated the role of mitochondrial SIRT3 in regulating macroautophagy and CMA, as well as modulating LDs dispersion, to ameliorate lipotoxicity in hepatocytes, which demonstrated that SIRT3 exerts indispensable role in protecting against NAFLD.

## Materials and methods

### Animals

Male C57BL/6J mice (aged 8–10 weeks) were purchased from the animal facility of Faculty of Health Sciences, University of Macau. The mice were randomly distributed into two groups, each five mice. The mice of RD group were fed a regular chow diet (Guangdong Medical Lab Animal Center, Guangzhou, Guangdong, China) and water *ad libitum*. The mice of HFD group were fed with a 45% high fat diet (Trophic Animal Feed High-Tech Co., Nantong, Jiangsu, China) for eighteen weeks to induce fatty liver. All mice were housed in a standard light (i.e., 12/12-h light/dark), temperature (21 ± 2 °C), and relative humidity (60 ± 10%) condition. The mice were sacrificed under isoflurane anesthesia. Liver samples of the middle of the right lobe were used for isolation of primary hepatocytes, and the remaining liver tissues were snap-frozen and stored at −80 °C for other studies. All procedures in animal experiments were approved by the Experimental Animal Ethics Committee at University of Macau (No. ICMS-AEC-2014-06). All procedures involved in the animal experiments were carried out in accordance with the approved guidelines and regulations.

### Isolation of primary hepatocytes

Primary hepatocytes were isolated using the two-step Percoll gradient method as described previously [17]. Liver was perfused with Ca^2+^ and Mg^2+^-free Hank’s buffered salt solution (HBSS, Gibco, Grand Island, NY, USA) containing EGTA (2.5 mM), and then digested with collagenase buffer containing collagenase type IV (0.5 mg/ml, Roche, Basel, Switzerland), NaCl (66.7 mM), KCl (6.7 mM), HEPES (50 mM), and CaCl_2_ (4.8 mM). Digested liver was dissected and then gently teased into small pieces with forceps. The liver slurry was filtered through a 100 μm nylon cell strainer (BD, Lake Franklin, NJ, USA). After spin down at 1200 g for 5 min, the cell pellet was resuspended with HBSS. Then the cell suspension was centrifuged at 400 g for 3 min to collect the pellet. The pellet was resuspended in 25% Percoll and centrifuged at 550 g for 5 min with the brake option off. The pellet was washed with PBS and then lysed with RIPA lysis buffer (Beyotime, Shanghai, China) containing 1% protease inhibitor cocktail and 1% phenylmethane sulfonylfluoride (Sigma-Aldrich, St. Louis, MO, USA) for immunoblotting.

### Cell culture and treatments

AML12 cells were purchased from American Type Culture Collection (Rockville, MD, USA), and cultured in DMEM supplemented with 10% FBS and ITS-G (5 mg/ml insulin, 5 mg/l transferrin, 5 μg/l selenous acid, Peiyuan Biotechnology, Shanghai, China) in humidified air containing 5% CO_2_ at 37 °C. Oleic acid and palmitic acid were completely dissolved in 75% (v/v) ethanol by heating at 55 °C, and then diluted in DMEM containing 1% (w/v) fatty acid-free bovine serum albumin (Sigma-Aldrich, A8806) to 500 μM and 250 μM, respectively. The solution was placed in shaker incubator for 2 h and then sterilized by passing through 0.2 μm filters to make P/O solution. For LD loading, AML12 cells were treated with P/O solution for 24 h. Then, cells were incubated in the standard culture medium (loaded) or the medium without FFA and glucose (supplemented with L-glutamine, P/S and non-essential amino acids, unloading) for 24 h, and in some cases with the indicated chemicals.

### Generation of SIRT3 overexpression cell line

The pcDNA3.1-SIRT3-Flag plasmid was generated as described previously [18]. The pcDNA3.1-SIRT3-Flag and pcDNA3.1 plasmids were transfected into AML12 cells using Lipofectamine 2000 (Invitrogen, Carlsbad, CA, USA) following manufacture’s instruction [19]. Briefly, AML12 cells (4 × 10^5^) were seeded at 35 mm plates. After 24 h, the cells were transfected with 10 μg plasmids (pcDNA3.1 or pcDNA3.1-SIRT3-Flag) using Lipofectamine 2000 reagent. 24 h after transfection, 800 μg/ml G418 (Sigma-Aldrich) was added to select positive cells for 12 days. Medium was changed every other day.

### RNA interference

The shRNA targeting *SIRT3* (mouse, sc-61556) and scrambled RNA (mouse, sc-108060), and shRNA transfection reagent (mouse, sc-108061) were purchased from Santa Cruz Biotechnology (Santa Cruz, CA, USA). AML12 cells were transfected with 2 μg shRNA for 6 h according to the manufacturer’s protocol. Cells were switched to fresh medium and incubated for an additional 24 h. Then, cells were selected with 2 μg/ml puromycin (Sigma-Aldrich) for 6 days, and then 4 μg/ml puromycin for 6 days. Thereafter, cells were pooled together for further experiments.The siRNA targeting *Atg5* (#1080, sense: GCUACCCAGAUAACUUUCUTT; antisense: AGAAAGUUAUCUGGGUAGCTT), *LAMP-2A* (#215, sense: GCCGUUCAGUCCAAUGCAUTT; antisense: AUGCAUUGGACUGAACGGCTT), *AMPKα1* (#1250, sense: GCCGACCCAAUGAUAUCAUTT; antisense: AUGAUAUCAUUGGGUCGGCTT), *AMPKα2* (#1059, sense: GCAGUGGCUUAUCAUCUUATT; antisense: UAAGAUGAUAAGCCACUGCTT), *AMPKβ1* (#610, sense: GCCAGCUUGGCACAGUUAATT; antisense: UUAACUGUGCCAAGCUGGCTT), and *AMPKβ2* (#608, sense: CCAGUCAGCUUGGAACAAUTT; antisense: AUUGUUCCAAGCUGACUGGTT) were purchased from GenePharma (Shanghai, China). Scrambled non-targeting siRNA was used as a negative control. AML12 cells (1 × 10^5^) were seeded at 6-well plates. After 24 h, the cells were transfected with 10 nM siRNA using Lipofectamine 3000 and OPTI-MEMI-reduced serum medium (Invitrogen) for 6 h. Cells were switched to fresh medium and incubated for an additional 24 h. Thereafter, cells were used for further experiments.

### Transient transfection and infection

The plasmid of pEGFP-LC3 (microtubule-associated protein 1 light chain 3) was purchased from Addgene (#21073, Cambridge, MA, USA). The plasmid of pCMV3-SCD1-N-Myc was purchased from Sino Biological (MG51311-NM, Wayne, PA, USA). The plasmid was transiently transfected into AML12 cells using Lipofectamine 2000 (Invitrogen) following manufacture’s instruction. Briefly, AML12 cells (2 × 10^5^) were seeded at 6-well plates. After 24 h, the cells were transfected with 5 μg plasmid using Lipofectamine 2000 reagent. 24 h after transfection, the cells were used for further experiments.

Ad-mCherry-GFP-LC3 was purchased from Beyotime (#C3011, Shanghai, China). AML12 cells (2 × 10^5^) were seeded on the coverslips in 6-well plates. After 24 h, the cells were infected with 10 μl Ad-mCherry-GFP-LC3 (multiplicity of infection = 5) for 6 h according to the manufacturer’s protocol. Cells were switched to fresh medium and incubated for an additional 24 h. Then, the cells were fixed and blocked for fluorescence detection.

### Cell viability assay

AML12 cells viability was determined using 3-(4,5-dimethylthiazol-2-yl)-2,5-diphenyltetrazolium bromide (MTT) assay as described previously [20]. Briefly, AML12 cells (1 × 10^4^ cells/well) were seeded into a 96-well plate, and cultured for 16 h. For P/O mixture treatment, the cells were treated with P/O mixture. After 24 h, 1 mg/ml MTT solution was added to each well and the 96-well plates were further incubated for 4 h at 37 °C. Subsequently, 100 μl DMSO was added to each well to solubilize the formazan precipitates. Absorbance at 570 nm was measured by a microplate reader (flexstation 3, Molecular Devices, CA, USA). The cell viability was expressed as percentage of the control cells.

### Nile red staining

Nile read staining was performed as described previously [21]. Briefly, AML12 hepatocytes was fixed with 10% formaldehyde solution and stained with nile red (1 μg/ml). After incubated for 30 min at 4 °C and then washed with PBS, cellular nile red-stained LDs were observed using fluorescence microscopy, and quantitated with flow cytometer with excitation and emission wavelength at 530 nm and 590 nm, respectively.

### Immunofluorescence staining

AML12 cells were seeded on the coverslips in 6-well plates. The cells were fixed in 10% formalin and then blocked with 2.5% goat serum. Primary antibodies were visualized with Texas Red-conjugated secondary antibodies (Thermo Fisher, Rockford, IL, USA). Cell nuclei were stained with DAPI or Hoechst 33258 (Sigma-Aldrich). The images were captured by the confocal fluorescence microscopy (Leica TCS SP8).

### Immunoblotting

Primary hepatocytes and AML12 cells were lysed with RIPA lysis buffer containing 1% protease inhibitor cocktail and 1% phenylmethane sulfonylfluoride. Protein concentration was determined using a BCA Protein Assay Kit (Pierce, Rockford, IL, USA). Equal amount of proteins were separated using 5–12% sodium dodecyl sulfate-polyacrylamide gel electrophoresis (SDS–PAGE), and then transferred to polyvinylidene fluoride membranes. After blocked with 5% nonfat milk for 2 h at room temperature, the membranes were probed with specific primary antibodies (Supplementary Table 1) overnight at 4 °C, and then probed with corresponding secondary antibodies for 1 h at room temperature. Signals were developed using a SuperSignal West Femto Maximum Sensitivity Substrate kit (Thermo, Rockford, IL, USA). Then, specific protein bands were visualized using the ChemiDoc MP Imaging System, and quantification was performed with Image Lab 5.1 (Bio-Rad, Hercules, CA, USA).

### Immunoprecipitation

Immunoprecipitation was performed as described previously [22]. Briefly, cell lysates (3 mg protein) were mixed with the indicated antibody (2 μg) at 4 °C overnight. Then 20 μl protein A/G-agarose beads (Santa Cruz) were added to the cell lysate and incubated on a rotator for 4 h at 4 °C. Subsequently, the beads were washed three times with PBS. Immune complexes were washed two times with lysis buffer supplemented with complete mini-protease inhibitor cocktail. Bound proteins were boiled in sample preparation buffer for 5 min and then used for immunoblotting.

### Lipid droplet isolation

LDs were isolated as described. Briefly, cells were scraped in PBS, lysed in hypotonic buffer (50 mM HEPES, 1 mM EDTA and 2 mM MgCl_2_, pH 7.4) supplemented with protease inhibitors with 50 strokes in a Dounce homogenizer. After spinning 5 min at 1500 g, post-nuclear fractions were mixed with equal volumes of 1.05 M sucrose in isotonic buffer (50 mM HEPES, 100 mM KCl, 2 mM MgCl_2_) and was centrifuged with 100,000 g for 2 h to remove Golgi, rough ER, mitochondria and peroxisomes. The supernatant was collected and adjusted to 1 M sucrose in hypotonic buffer and layered on a sucrose gradient with 1 ml of each 0.75, 0.5, 0.25, 0.125, 0 M sucrose. The sucrose gradient tube was centrifuged at 100,000 g at 4 °C for 4 h. LD fractions were collected from the top of the tube and delipidated with acetone followed by sequential washes in acetone/ether (1:1, v:v). The Pellet was dried under a gentle stream of nitrogen and re-suspended in protein lysis buffer. Protein concentration of LD fractions were determined by BCA Protein Assay Kit. Equal amounts of total protein were subjected to SDS–PAGE for Western blotting analysis.

### Mitochondrial membrane potential (ΔΨm) assay

The ΔΨm was determined by the fluorescent dye Rhodamine123, a cell-permeable cationic dye that preferentially partitions into mitochondria based on the highly negative ΔΨm. Briefly, AML12 cells were treated as described in cell viability section. Then cells were stained with Rhodamine123 (10 μM) for 10 min. Subsequently, cells were washed twice with PBS, trypsinized and collected in a 1.5 ml tube. The change of ΔΨm was qualitatively observed on an In Cell Analyzer 2000 (GE Healthcare Life Sciences, Chicago, IL, USA).

### Isoproterenol-induced lipolysis

The lipolysis activity of AML12 cells was measured as describe previously [23]. Briefly, the cells were incubated at 37 °C with 10 μM isoproterenol (Sigma-Aldrich) as stimulated condition, or DMSO as basal condition. 2 h later, medium was collected and heated at 85 °C for 10 min. After spin down, clear supernatant was transferred to a new tube, and 10 μl was used to measure free glycerol content using Free Glycerol Reagent (Sigma-Aldrich). Lipolysis activity was represented by glycerol concentrations, normalized by protein concentration.

### Seahorse analysis

A Seahorse Bioscience XF24-3 Extracellular Flux Analyzer (Agilent, Santa Clara, CA) was used to measure the oxygen consumption rate (OCR) as described previously [24]. AML12 were seeded in XF24-well microplates (Seahorse Bioscience, Billerica, MA) at 5 × 10^4^ cells per well. The cells were treated with or without P/O mixture for 24 h. Then, the cells were incubated in the absence of CO_2_ for 1 h. XF assay medium was low-buffered bicarbonate-free DMEM (pH 7.4) and replicated the glucose and pyruvate/glutamax composition of the respective experimental conditions. After measuring basal OCR, 1 μM carbonyl cyanide-p-trifluoromethoxyphenylhydrazone (FCCP, Sigma-Aldrich) were introduced in real time. After detection, cellular protein content was quantitated with a BCA kit and OCR were normalized accordingly.

### Statistical analysis

Data were analyzed using GraphPad Prism-6 (GraphPad Software, San Diego, CA). All experimental data were expressed as mean ± S.D., and sample size for each experiment corresponds to three biological replicates. Significant differences between groups were determined using a one-way analysis of variance (ANOVA) with Dunnet’s multiple comparisons test, considering *P* < 0.05 as significant differences. Where statistical significance is evaluated, variance between groups is confirmed to be similar between comparison groups (control vs. experimental) and the statistical analysis is deemed appropriate.

## Results

### Over nutrition induced lower SIRT3 expression and impaired macroautophagy in hepatocytes in vitro and in vivo

**Fig. 1:**
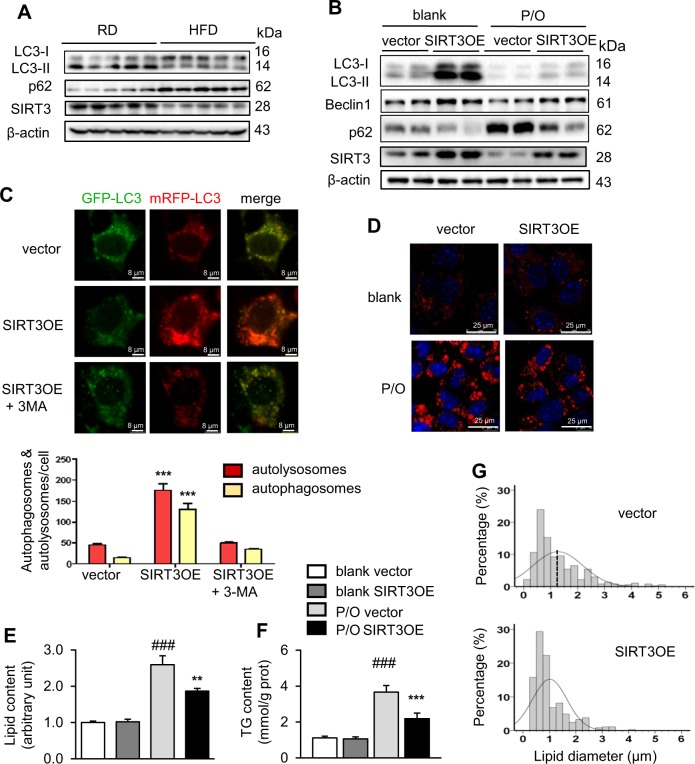
SIRT3 overexpression activates macroautophagy and attenuates lipid content in lipotoxic hepatocytes. **a** LC3, p62 and SIRT3 protein levels in primary hepatocytes from RD- and HFD-fed mice. The liver samples were collected from mice with the age of 26‒28 weeks, fed with either RD or HFD for 18 weeks. *n* = 5. **b** LC3, Beclin1, p62 and SIRT3 protein levels in both vector and SIRT3OE AML12 hepatocytes treated with or without P/O mixture for 24 h. **c** SIRT3OE and vector AML12 cells were infected with the Ad-mCheryy-GFP-LC3. The mRFP-LC3 and GFP-LC3 puncta were examined by using a confocal microscope. Scale bar = 8 μm. The numbers of autophagosomes (yellow puncta) and autolysosomes (red puncta) were quantified. **d** Intracellular lipid content was visualized with nile red (red) staining. Nucleus were stained with Hoechst 33258 (blue). Scale bar = 25 µm. **e** The lipid content was determined by nile red fluorescence using flow cytometry. White: blank, vector; gray: blank, SIRT3OE; light gray: P/O, vector; black: P/O, SIRT3OE. **f** The cellular TG content. White: blank, vector; gray: blank, SIRT3OE; light gray: P/O, vector; black: P/O, SIRT3OE. **g** The distribution of LDs diameter. Data are shown as mean ± S.D., *n* = 6, **p < 0.01 and ***p < 0.001, vector vs. SIRT3OE. ###*p* < 0.001, blank vs. P/O treatment. One-way ANOVA was used to calculate the *p*-values

Macroautophagic defects and SIRT3 deletion are positively correlated with ectopic lipid deposition [14, 25]. Previous reports showed that HFD-fed mice express lower SIRT3 protein in liver [26, 27]. Herein, we confirmed that the primary hepatocytes from HFD-fed mice expressed lower SIRT3 protein, compared with those from RD-fed mice (Fig. 1a). In the process of macroautophagy, LC3 is cleaved by ATG4 to generate the cytoplasmic form LC3-I, which is subsequently converted into the active form LC3-II through conjugating with phosphatidylethanolamine [28]. The ratio of LC3-II/LC3-I is commonly used as a marker of macroautophagy activation. The primary hepatocytes from HFD-fed mice exhibited impaired macroautophagy, indicating by reduced ratio of LC3-II/LC3-I and accumulated macroautophagic substrate p62, compared with those from RD-fed mice (Fig. 1a). It suggested that SIRT3 and macroautophagy might play a protective role against lipotoxicity in hepatocytes.

Palmitic and oleic acids are widely used to induce steatosis in culture hepatocytes [29, 30]. Mixture of oleic and palmitic acids induced the same extent of steatosis but lower cytotoxicity than did palmitic acid alone [30]. Thus, a mixture of oleic and palmitic acids with a ratio of 1:2 (P/O) was used to induce lipid accumulation in murine hepatic AML12. Consistently, the protein levels of SIRT3 and Beclin1, and ratio of LC3-II/LC3-I were decreased, and the protein level of p62 was increased in P/O-treated AML12 cells (Fig. 1b). These data suggested that over nutrition induced lower SIRT3 expression and impaired macroautophagy in hepatocytes.

### SIRT3 overexpression attenuates lipid accumulation in lipotoxic hepatocytes through promoting macroautophagy

To further evaluate the role of SIRT3 in protecting hepatocytes against lipotoxicity, a SIRT3 overexpressed AML12 cell line (SIRT3OE) was generated using a transgene in which the Flag-tag fused to C-terminus of the full-length SIRT3 cDNA. As expected, gain of SIRT3 expression exerted profound effect on activation of macroautophagy in P/O-treated hepatocytes, indicating by increases of Beclin1 protein level and LC3-II/LC3-I ratio, and a reduction of p62 protein level, compared to the vector cells (Fig. 1b). To further evaluate the macroautophagic flux, the mRFP-LC3 and GFP-LC3 puncta colocalization was assessed in SIRT3OE cell infected with an mCherry-GFP-LC3 tandem construct. GFP fluorescence is stable only in autophagosomes and can be quenched easily in acidic environments such as autolysosomes, whereas mRFP is more stable under acidic conditions and can be detected in both autophagosomes and autolysosomes [31]. In SIRT3 overexpressed cells, more red-only puncta were observed (Fig. 1c), indicating that the macroautophagic flux was enhanced in SIRT3OE cells without disrupting the lysosomal function and/or autophagosome-lysosome fusion. When treated with the macroautophagic inhibitor, 3-methyladenine (3-MA), the red-only puncta in SIRT3OE cells was obviously reduced (Fig. 1c). These results indicated that the macroautophagic flux was enhanced in SIRT3OE hepatocytes under lipotoxic status.

Intriguingly, P/O treatment induced about a 2.5-fold increase of lipid content, and SIRT3 overexpression attenuated this trend, indicating by the qualitative and quantitative analyses of nile red staining (Fig. 1d, e). P/O-induced increase of intracellular triglycerides (TGs), but not total cholesteral, in AML12 cells was reversed by SIRT3 overexpression (Fig. 1f, Supplementary Figure S1b). Autophagy-deficient hepatocytes are characterized by not only increased lipid content but also increased LD size and number [5]. SIRT3 overexpression-induced activation of macroautophagy might affect LD size in P/O-treated hepatocytes. The results indicated that the mean diameter of LDs in SIRT3OE cells (1.01 ± 0.66 μm) was smaller than the vector cells (1.27 ± 0.92 µm) (Fig. 1g).

**Fig. 2:**
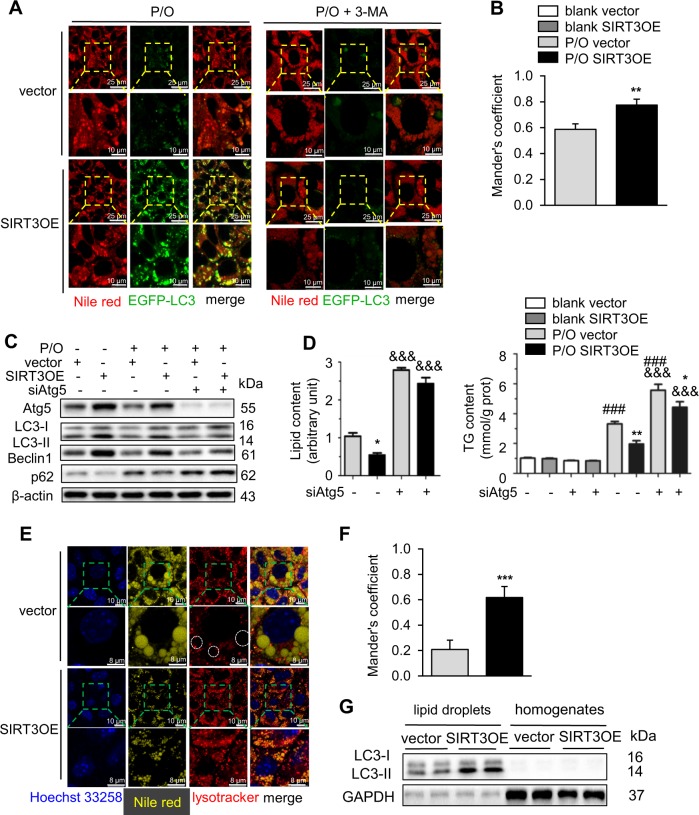
SIRT3 protects hepatocytes against lipotoxicity through enhancing macroautophagy in LDs. **a** The SIRT3OE cells induced more EGFP-LC3 puncta on LDs; and 3-MA treatment totally blocked it. LDs were visualized with nile red (red) staining. Scale bar = 25 or 10 µm. **b** The Mander’s overlapping coefficient of EGFP-LC3 and nile red. Light gray: P/O, vector; black: P/O, SIRT3OE. **c** Knockdown of *Atg5* reversed SIRT3-induced activation of macroautophagy. Atg5, LC3, Beclin1 and p62 protein levels were detected using Western blots. **d** The lipid content and the cellular TG content in vector and SIRT3OE hepatocytes treated with or without si*Atg5*. White: blank, vector; gray: blank, SIRT3OE; light gray: P/O, vector; black: P/O, SIRT3OE. **e** SIRT3 overexpression enhanced the co-localization of lysosomes and LDs in P/O treated hepatocytes. Cells were stained with lysotracker (red), nile red (yellow) and Hoechst 33258 (blue). Scale bar = 10 µm. **f** The Mander’s overlapping coefficient of lysotracker and nile red. Light gray: P/O, vector; black: P/O, SIRT3OE. **g** SIRT3 overexpression increased the ratio of LC3-II/LC3-I mainly in LDs. Data are shown as mean ± S.D., *n* = 6, *p < 0.05, **p < 0.01 and ***p < 0.001, vector vs. SIRT3OE. ###*p* < 0.001, blank vs. P/O treatment. &&&*p* < 0.001, scrambled vs. si*Atg5*. One-way ANOVA was used to calculate the *p*-values

Next, SIRT3 overexpression enhanced autophagosome formation, which was largely co-localized with LDs (Fig. 2a). The Mander’s overlap coefficient factor of SIRT3OE cells were significantly higher than that of the vector cells (Fig. 2b). 3-MA treatment resulted in more severe lipid accumulation, and almost abolished SIRT3-mediated reduction of lipid accumulation in hepatocytes (Fig. 2a). On the other hand, pharmacological activation of macroautophagy with rapamycin reduced P/O induced lipid accumulation in hepatocytes (Supplementary Figure S1c). 3-MA is a non-specific intervention to block macroautophagy. The role of macroautophagy in SIRT3-mediated amelioration of lipotoxicity was further evaluated with *Atg5* knockdown. SiRNA-mediated *Atg5* knockdown almost blocked SIRT3-induced activation of macroautophagy, and reduction of lipid and TG contents in P/O-treated hepatocytes (Fig. 2c, d).

SIRT3 overexpression obviously enhanced the co-localization of lysosomes with LDs (Fig. 2e), which was further confirmed with the higher Mander’s overlap coefficient factor in SIRT3OE cells (Fig. 2f). Furthermore, gain of SIRT3 expression increased the ratio of LC3-II/LC3-I, which was mainly in LDs (Fig. 2g). Taken together, macroautophagy is involved in the protective effect of SIRT3 in ameliorating lipotoxicity in hepatocytes.

### SIRT3 promotes macroautophagy in lipotoxic hepatocytes through AMPK-ULK1 pathway

**Fig. 3:**
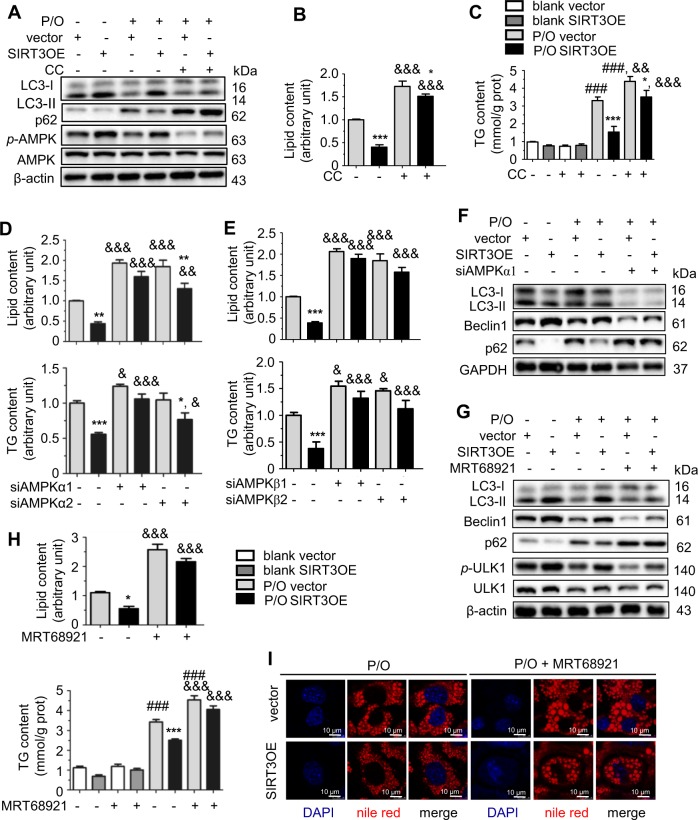
SIRT3 activates macroautophagy in lipotoxic hepatocytes through AMPK-ULK1 pathway. **a** LC3, p62, phosphorylated AMPK, and AMPK protein levels in vector and SIRT3OE hepatocytes treated with or without CC. The lipid content **b** and the cellular TG content **c** were determined in vector and SIRT3OE hepatocytes treated with or without CC. White: blank, vector; gray: blank, SIRT3OE; light gray: P/O, vector; black: P/O, SIRT3OE. &&*p* < 0.01, DMSO vs. CC. **d** The lipid content and the cellular TG content were determined in vector and SIRT3OE hepatocytes with silenced *AMPKα1* or *AMPKα2*. &*p* < 0.05, &&*p* < 0.01, and &&&*p* < 0.001, scrambled vs. si*AMPKα1*/si*AMPKα2*. Light gray: P/O, vector; black: P/O, SIRT3OE. **e** The lipid content and the cellular TG content were determined in vector and SIRT3OE hepatocytes with silenced *AMPKβ1* or *AMPKβ2*. Light gray: P/O, vector; black: P/O, SIRT3OE. &*p* < 0.05, &&*p* < 0.01, and &&&*p* < 0.001, scrambled vs. si*AMPKβ1*/si*AMPKβ2*. **f** LC3, Beclin1, and p62 protein levels in vector and SIRT3OE hepatocytes with silenced *AMPKα1*. **g** LC3, Beclin1, p62, phosphorylated ULK1, total ULK1 and β-actin protein levels in vector and SIRT3OE hepatocytes treated with or without MRT68921. **h** The lipid content and the cellular TG content in vector and SIRT3OE hepatocytes treated with or without MRT68921. White: blank, vector; gray: blank, SIRT3OE; light gray: P/O, vector; black: P/O, SIRT3OE. &&&*p* < 0.001, DMSO vs. MRT68921. **i** Intracellular lipid in vector and SIRT3OE hepatocytes treated with or without MRT68921 was visualized with nile red (red) staining. Nucleus were stained with DAPI (blue). Scale bar, 10 µm. Data are shown as mean ± S.D., *n* = 6, *p < 0.05, **p < 0.01 and ***p < 0.001, vector vs. SIRT3OE. ###*p* < 0.001, blank vs. P/O treatment. One-way ANOVA was used to calculate the *p*-values

AMPK orchestrates macroautophagy through increasing transcription of macroautopahgy-related genes and directly activating proteins involved in the initiation and nucleation steps of macroautophagy [32]. SIRT3 overexpression increased the phosphorylation of AMPK in P/O-treated hepatocytes, whereas SIRT3 silencing exhibited opposite results (Fig. 3a, Supplementary Figure S2a). Treatment of AMPK inhibitor, Compound C (CC), resulted in more lipid and TG accumulation in both SIRT3OE and vector cells (Fig. 3b, c, and Supplementary Figure S2b). CC treatment almost reversed the effect of SIRT3 on activation of macroautophagy (Fig. 3a), and attenuation of lipid and TG (Fig. 3b, c). Intriguingly, the AMPK activator 5-aminoimidazole-4-carboxamide ribonucleotide (AICAR), in turn, increased SIRT3 expression (Supplementary Figure S2c), reduced lipid and TG accumulation in P/O-treated AML12 cells, and almost abolished SIRT3 silencing induced lipid accumulation (Supplementary Figure S2d, e). AMPK exists as a trimeric complex consisting of a catalytic subunit (α subunit) and two regulatory subunits (β and γ subunits) [33]. AMPKα1, AMPKβ1, and AMPKγ1 are ubiquitously expressed, while, AMPKα2 and AMPKβ2 are expressed in the liver at relatively low level. Thus, we performed siRNA-mediated knockdown of *AMPKα1*, *AMPKα2*, *AMPKβ1* and *AMPKβ2* on vector and SIRT3OE cells, with the knockdown efficiencies ranging from 68% to 78% (Supplementary Figure S3). Knockdown of *AMPKα1*, but not* AMPKα2*, totally abolished the effect of SIRT3 in attenuating lipid and TG contents (Fig. 3d). On the other hand, knockdown of either *AMPKβ1* or *AMPKβ2* almost blocked the effect of SIRT3 (Fig. 3e). Moreover, knockdown of *AMPKα1* totally reversed SIRT3-induced activation of macroautophagy (Fig. 3f). Taken together, SIRT3 promotes macroautophagy and attenuates lipid accumulation in P/O-treated hepatocytes through activating AMPK.

AMPK directly phosphorylates and activates unc-51-like kinase 1 (ULK1) to induce macroautophagy [34, 35]. Indeed, SIRT3 overexpression increased the phosphorylated ULK1 level in P/O-treated hepatocytes (Fig. 3g). Treatment of MRT68921, a potent ULK1 inhibitor, totally blocked SIRT3-induced activation of macroautophagy, indicating by decreased LC3-II/LC3-I ratio and Beclin1 level, and increased p62 level (Fig. 3g). As expected, MRT68921 treatment dramatically increased lipid and TG contents in P/O-treated hepatocytes, and almost abolished the effects of SIRT3 on lipid and TG contents (Fig. 3h, i). Taken together, SIRT3 promotes macroautophagy to attenuate lipid accumulation in hepatocytes through AMPK-ULK1 pathway.

### SIRT3 promotes CMA in lipotoxic hepatocytes through activating AMPK

**Fig. 4:**
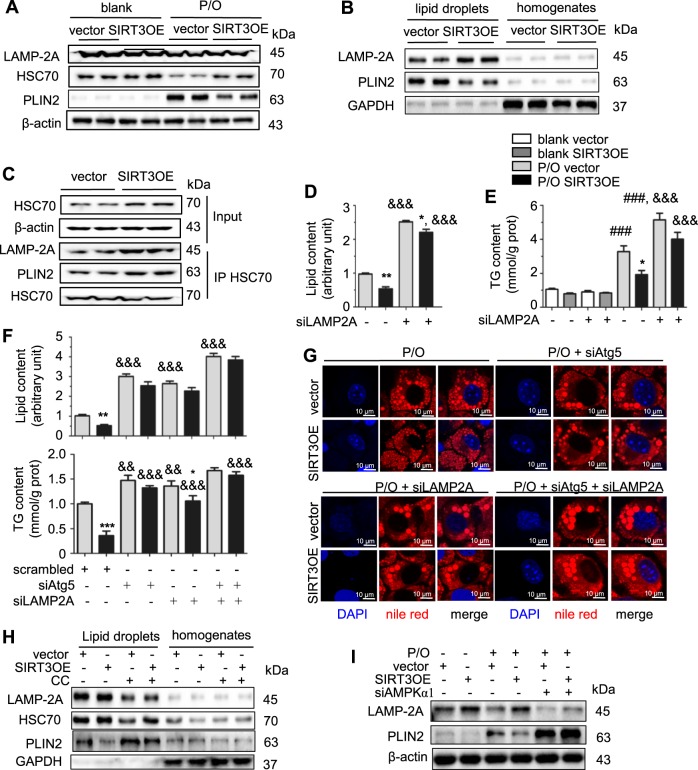
SIRT3 promotes CMA to attenuate lipid accumulation in P/O-treated hepatocytes. **a** LAMP-2A, HSC70 and PLIN2 protein levels in vector and SIRT3OE hepatocytes treated with or without P/O mixture for 24 h. **b** LAMP2 and PLIN2 levels in LDs and homogenates from vector and SIRT3OE cells. **c** LAMP-2A and PLIN2 proteins co-immunoprecipitated with HSC70 were examined. The lipid content **d** and the cellular TG content **e** were determined in vector and SIRT3OE hepatocytes treated with srambled siRNA or si*LAMP-2A*. White: blank, vector; gray: blank, SIRT3OE; light gray: P/O, vector; black: P/O, SIRT3OE. &&&*p* < 0.001, scrambled vs. si*LAMP-2A*. **f** The lipid content and the cellular TG content were determined in vector and SIRT3OE hepatocytes with silenced *Atg5*, silenced *LAMP-2A* or simultaneously silenced *Atg5/LAMP-2A*. Light gray: P/O, vector; black: P/O, SIRT3OE. &&&*p* < 0.001, scrambled vs. si*LAMP-2A*, si*Atg5* or si*LAMP-2A*/si*Atg5*. **g** Intracellular lipid in vector and SIRT3OE hepatocytes with silenced *Atg5*, silenced *LAMP-2A*or simultaneously silenced *Atg5*/*LAMP-2A*was visualized with nile red (red) staining. Nucleus were stained with DAPI (blue). Scale bar = 10 µm. **h** LAMP-2A, HSC70 and PLIN2 protein levels in LDs and homogenates from cells treated with or without CC. **i** LAMP-2A and PLIN2 protein levels in vector and SIRT3OE cells treated with srambled siRNA or si*AMPKα1*. Data are shown as mean ± S.D., *n* = 6, *p < 0.05, **p < 0.01 and ***p < 0.001, vector vs. SIRT3OE, ###*p* < 0.001 blank vs. P/O treatment. One-way ANOVA was used to calculate the *p*-values

CMA is required for the initiation of lipolysis by either cytosolic lipases or macroautophagy [9]. SIRT3 might regulate CMA to induce the degradation of LDs in P/O-treated hepatocytes. Treatment of P/O did not obviously affect LAMP-2A expression, but suppressed HSC70 level and increased PLIP2 level (Fig. 4a). Interestingly, SIRT3 overexpression obviously increased HSC70 level and suppressed PLIN2 level, but did not affect LAMP-2A level in P/O-treated hepatocytes, compared to those of the vector cells (Fig. 4a). In isolated LDs, gain of SIRT3 expression increased LAMP-2A level and decreased PLIN2 level (Fig. 4b). Consistently, knockdown of SIRT3 exhibited opposite results in LDs (Supplementary Figure S4a). When pulled down HSC70, both LAMP-2A and PLIN2 proteins were obviously increased in SIRT3OE hepatocytes compared to those in the vector cells (Fig. 4c), which indicated that SIRT3 overexpression promoted CMA process and enhanced the degradation of PLIN2 in LDs. To verify that SIRT3 attenuates lipid accumulation through activating CMA, LAMP-2A was knocked-down in vector and SIRT3OE cells (Supplementary Figure S4b). As expected, knockdown of *LAMP-2A* almost abolished SIRT3’s effect on reducing lipid and TG contents in P/O-treated hepatocytes (Fig. 4d, e). Furthermore, simultaneous knockdown of *Atg5* and *LAMP-2A* induced more severe lipid accumulation compared with either *Atg5* knockdown or *LAMP-2A* knockdown, and almost abolished the SIRT3’s effect on reduction of lipid accumulation in P/O-treated AML12 cells (Fig. 4f, g). These results indicated that the effect of SIRT3 on amelioration of lipotoxicity in hepatocytes is attributed to both macroautophagy and CMA.

Activation of AMPK promotes CMA to induce LD breakdown [9]. The immunoblot analysis showed that CC treatment almost reversed the increases of LAMP-2A and HSC70 levels, and the decrease of PLN2 level in LDs from SIRT3OE cells (Fig. 4h). In agreement, knockdown of *AMPKα1* totally blunted SIRT3-indcued increase of LAMP-2A and decrease of PLN2 in P/O treated cells (Fig. 4i). Taken together, SIRT3 promotes CMA to attenuate lipid accumulation in hepatocytes through activating AMPK.

### SIRT3 suppresses SCD1 expression in lipotoxic hepatocytes

**Fig. 5:**
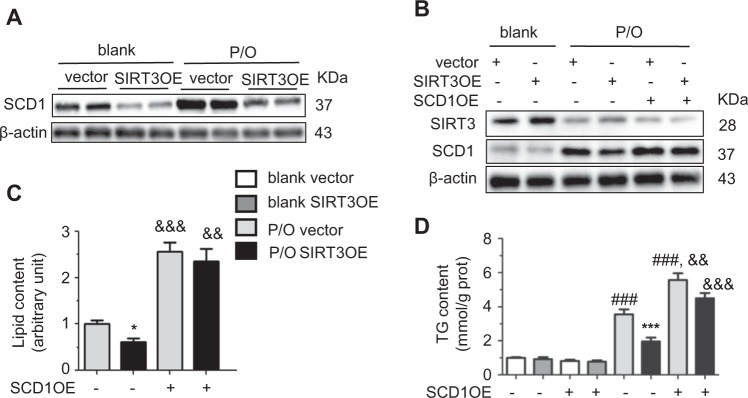
SIRT3 suppresses lipogenesis in lipotoxic hepatocytes. **a** SCD1 protein level in vector and SIRT3OE hepatocytes treated with or without P/O mixture for 24 h. **b** SIRT3 and SCD1 protein levels in SIRT3 and SCD1 double overexpressed hepatocytes. The lipid content **c** and the cellular TG content **d** in SIRT3 and SCD1 double overexpressed hepatocytes. White: blank, vector; gray: blank, SIRT3OE; light gray: P/O, vector; black: P/O, SIRT3OE. Data are shown as mean ± S.D., *n* = 6, *p < 0.05, and ***p < 0.001, vector vs. SIRT3OE, ###*p* < 0.001 blank vs. P/O treatment. && *p* < 0.01, &&& *p* < 0.001, SCD1OE vs. vector. One-way ANOVA was used to calculate the *p*-values

Stearoyl-CoA desaturase 1 (SCD1) is a key lipogenic enzyme that catalyzes the rate-limiting step in the formation of monounsaturated long-chain acyl CoAs from saturated long-chain acyl CoAs [36]. Mice with hyperlipidemia or humans with obesity and type II diabetes have increased SCD1 expression [37, 38]. SCD1 is induced in the liver of SIRT3 knockout mice through elevated saturated lipids, and HFD-induced metabolic complications observed in SIRT3 knockout mice are attenuated in SIRT3/SCD1 double knockout mice [27]. As expected, SCD1 expression was increased in P/O-treated AML12 cells, and gain of SIRT3 expression suppressed the SCD1 level (Fig. 5a), whereas SIRT3 silencing exhibited opposite results (Supplementary Figure S5). To identify the role of SCD1 down-regulation in SIRT3OE hepatocytes, a SIRT3 and SCD1 double overexpressed AML12 cell line was generated. Under P/O challenge, SCD1 overexpression suppressed the SIRT3 level (Fig. 5b). As expected, SCD1 overexpression increased lipid accumulation and totally abolished the effects of SIRT3 on lipid and TG contents in P/O-treated hepatocytes (Fig. 5c, d). Thus, SIRT3 rescues hepatocytes from P/O-induced lipotoxicity partially through suppressing SCD1-mediated lipogenesis.

### SIRT3 promotes LDs dispersion on detyrosinated microtubules to increase mitochondrial energetics

**Fig. 6:**
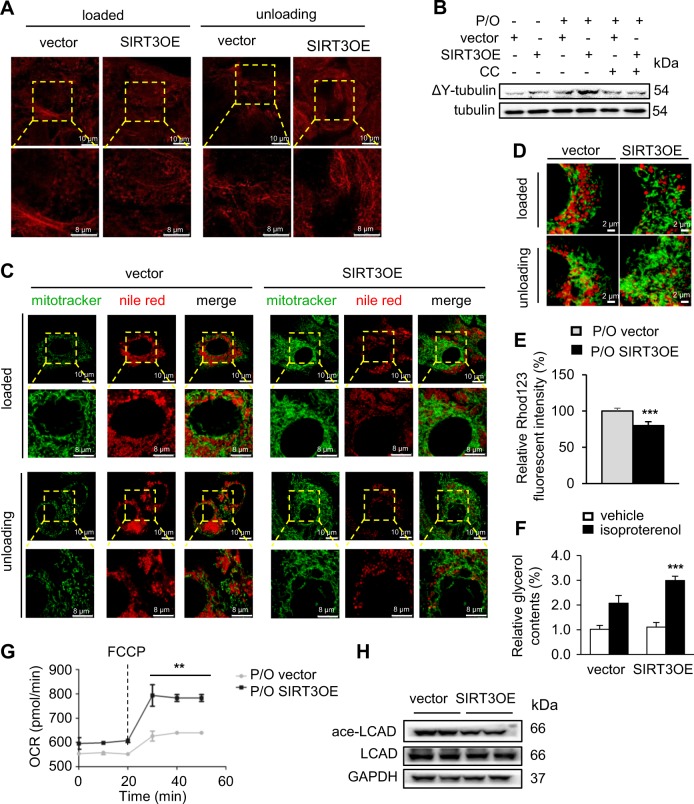
SIRT3 reorganized the network of microtubules to facilitate consumption of lipids. **a** Confocal images of loaded and unloading cells labeled with detyrosinated tubulin (red). Scale bar, 10 and 8 μm. **b** ΔY-tubulin and tubulin levels. **c** Nile red (red) and mitotracker (green) images of loaded and unloading vector and SIRT3OE cells. Scale bar = 10 and 8 µm. **d** 3D-rendering (LAS-X, Leica) of a confocal image stack of cells. Cells were stained with Nile red (red) and mitotracker (green). Scale bar = 2 μm. **e** The relative Rhod123 fluorescent intensity. Light gray: P/O, vector; black: P/O, SIRT3OE. **f** Relative glycerol content in hepatocytes under both normal and isoprenaline-induced conditions. White: vehicle; black: isoproterenol. **g** Mitochondrial OCR level under baseline and FCCP induction condition. Gray dot: P/O, vector; black square: P/O, SIRT3OE. **h** The acetylated and total LCAD levels. Data are shown as mean ± S.D., *n* = 6, **p < 0.01 and ***p < 0.001, vector vs. SIRT3OE. One-way ANOVA was used to calculate the *p*-values

During nutrient deprivation, LDs and mitochondria relocate on detyrosinated microtubules (MTs) to adopt a dispersed distribution through activation of AMPK [39]. In the cell periphery, LDs with a remarkable dispersion render stronger LD–mitochondria interactions and more efficient FFAs supply for mitochondrial β-oxidation. Herein, the vector and SIRT3OE cells were treated with P/O mixture for 24 h, and then maintained in standard medium (loaded) or medium without FFA and glucose (unloading) for additional 24 h. SIRT3 overexpression obviously reorganized the network of detyrosinated MTs during unloading state (Fig. 6a). SIRT3 overexpression induced an increase of ΔY-tubulin; while treatment of CC highly abrogated this trend (Fig. 6b). Under loaded status, the size of LDs appeared relatively constant, and the LDs were clumped and formed clusters around the nucleus in vector cells; in contrast, the LDs were dispersed in more perinuclear regions after unloading in the vector cells (Fig. 6c and Supplementary Figure S6a). Under unloading state, the LDs in SIRT3OE hepatocytes existed less in clusters and more in perinuclear regions (Fig. 6c). Owing to the complex three-dimensional (3D) organization of the MTs network, the interaction between organelles and the MTs was further examined by analyzing confocal microscopy images. The 3D reconstruction further confirmed the above results (Fig. 6d).

Rhodamine 123 is a green-fluorescent dye that is preferentially partitions into the mitochondrial based on the highly negative membrane potential without cytotoxic effects [20]. Rhodamine 123 staining results showed P/O treatment interrupted mitochondrial membrane potential (ΔΨm); while, SIRT3OE cells possessed higher ΔΨm, indicating improved mitochondrial function (Fig. 6e). SIRT3 overexpression stimulated lipolysis under isoproterenol treated condition, but not basal state (Fig. 6f). Using the Seahorse XFe analyzer, we found SIRT3 overexpression enhanced maximum oxygen consumption rate (OCR, Fig. 6g). Long-chain acyl-CoA dehydrogenase (LCAD), a key fatty acid β-oxidation enzyme, is a deacetylating substrate of SIRT3 [14]. SIRT3 overexpression decreased the acetylated LCAD level, but didn’t change the total LCAD expression (Fig. 6h). Consistently, SIRT3 knockdown cells showed higher acetylated LCAD level (Supplementary Figure S6b). Taken together, SIRT3 rescues hepatocytes from P/O mixture induced lipid accumulation by increasing LDs dispersion and mitochondrial energetics.

## Discussion

The current study showed that SIRT3 expression is negatively correlated with lipotoxicity in P/O-treated AML12 hepatocytes and primary hepatocytes from HFD-fed mice. It has been indicated that HFD feeding leads to lower peroxisome proliferator-activated receptor γ coactivator 1-α (PGC-1α) expression, which is responsible for the reduced SIRT3 expression, and overexpression of PGC-1α rescues the phenotype [27]. A sequence motif in the SIRT3 promoter is recognized by the estrogen related receptor-α (ERRα), and PGC-1α mediates ERRα binding to this sequence motif in the SIRT3 promoter and promotes SIRT3 gene expression [40]. In fact, PGC-1α expression was decreased in the primary hepatocytes from HFD-fed mice (Supplementary Figure S7), which might explain how lipotoxicity negatively regulates the SIRT3 expression.

Our and others studies indicated that the murine SIRT3 gene expresses three isoforms, the two long forms of murine SIRT3 proteins (M1 and M2) exist in mitochondria, while the short form of SIRT3 protein (M3) lacks an N-terminal mitochondrial targeting signal, mainly existing in cytosol [11–13]. Although the long and short forms of SIRT3 localize at different cellular compartments, all of them exhibit deacetylase activity on different substances [10, 41, 42]. Besides the full length SIRT3 (M1), the cytosolic SIRT3-M3 was found to protect hepatocytes against lipotoxicity (Supplementary Figure S8). It suggested that both mitochondrial and cytosolic SIRT3 protects hepatocytes against lipotoxicity.

SIRT3 has several distinct functional domains, such as metal binding site, catalytic site, and nucleotide binding site [43]. In our previous studies, an enzymatically inactive mutant SIRT3-M3 (N87A) totally abolished SIRT3’s effect on mitochondrial functions in brown adipocytes [44]. Moreover, an enzymatically inactive SIRT3 (H248Y) failed to deacetylate LCAD or regulate mitochondrial fatty acid oxidation [14]. The enzymatically inactive SIRT3 mutants, either SIRT3-M3 (N87A) or SIRT3 (H248Y), failed to activate AMPK or reduce lipid and TG accumulation in P/O-treated hepatocytes (Supplementary Figure S8). It suggested that the enzymatic activity of SIRT3 is absolutely required for its protective effects against lipotoxicity in hepatocytes.

Macroautophagy accounts for a high percentage of lipolysis in liver. Thus, blockage of macroautophagy through knockdown of the essential macroautophagic genes like *Atg* in hepatocytes, led to a significant increasing of LDs in cells even under normal nutritional conditions [5]. Interestingly, an increase in macroautophagic activity was detected during the first few weeks, which was followed by a gradual decrease in macroautophagy, in long-term HFD treated animals. The decrease of macroautophagy further exacerbated LDs accumulation, eventually leading to hepatotoxicity and severe steatosis [5, 45, 46]. Most previous studies have recognized SIRT3 as a positive regulator of macroautophagy [16, 47–50]. Recently, Li et al. reported that SIRT3 acted as a negative regulator of macroautophagy [51]. In their study, saturated fatty acids (SFAs) was used to induce lipotoxicity in both mice and hepatocytes. SFAs were reported to inhibit fusion between autophagosomes and lysosomes and decrease degradation of autophagosomes, leading to autophagosome accumulation [52]. Thus, SFAs might not induce macroautophagy indeed, but block macroautophagic flux. On the other hand, palmitic acid exhibited a dose-dependent cytotoxic effect associated with reactive oxygen species production, presented markers of apoptosis and necrosis and a decrease in albumin production [29, 30]. Therefore, SFAs induced apoptosis and/or necrosis, but not lipotoxicity, might result in the controversial observations in Li’s study. In contrast with palmitic acid, oleic acid induced a damage of the cytoplasmic membrane only at high concentration (1 mM and above). Mixture of oleic acid and palmitic acid induced lower cytotoxicity with less weakened functional capacity than did palmitic acid alone [29, 30].

AMPK is responsible for monitoring cellular energy status and is regulated through phosphorylation at amino acid residue threonine 172 [53]. It has been recognized that AMPK directly stimulates mitochondrial energy production and strengthens mitochondrial biogenesis. AMPK also drives macroautophagy through multiple mechanisms [32, 54]. AMPK directly phosphorylates and activates proteins involved in the initiation and nucleation steps of macroautophagy, such as ULK1 and Beclin1 [35, 55]. Furthermore, AMPK phosphorylates the transcription factor forkhead box O-3 (FoxO3), leading to increased transcription of macroautophagy-related genes [56]. SIRT3 deacetylates and activates liver kinase B1 (LKB1) which, in turn, stimulates AMPK activation [57, 58]. Alternatively, SIRT3 promotes an increase of cytosolic calcium level, which activates calcium/calmodulin-dependent kinase II (CaMKII) to phosphorylate AMPK [59]. Thus, SIRT3 induced macroautophagy in lipotoxic hepatocytes through SIRT3-AMPK-ULK1 pathway. Moreover, AMPK-dependent phosphorylation of PLIN2, a gatekeeper to keep LDs away from breakdown, triggers degradation of PLIN2, which is the first step to initiate lipolysis via either cytosolic lipases or macroautophagy [9]. SIRT3 enhanced CMA to induce LDs breakdown, through activation of AMPK. SIRT3 might be a key regulator in CMA mediated LDs breakdown and macroautophagy mediated LDs digestion.

Lipotoxicity, resulting from the accumulation of lipid intermediates in non-adipose tissue, causes cellular dysfunction and death but also initiates cellular stress response. SIRT3 deacetylates many enzymes involved in the responses to oxidative stress and nutrient deprivation. In the liver, SIRT3 regulates mitochondrial electron transport chain, such as complex I subunit NDUFA9 [60], complex II succinate dehydrogenase [61], and ATP synthase ATP5A [62]. Previous studies disclosed that SIRT3 is involved in promoting mitochondrial biogenesis and correcting mitochondrial structure defects [63, 64]. SIRT3 directly deacetylates LCAD to promote fatty acid β-oxidation [14]. It has been reported that SCD1 is induced in the liver of SIRT3 knockout mice through elevated saturated lipids [27]. Our studies showed SIRT3 protects hepatocytes from oxidative injury by enhancing ROS scavenging and mitochondrial integrity [65]. Many studies have revealed the protective effects of SIRT3 against lipotoxicity in diverse tissues, including liver. Although Li et al. claimed that SIRT3 enhanced the susceptibility of hepatocytes to SFAs-induced cytotoxicity, it should be pointed out that SFAs-induced lipotoxicity caused negligible cell death, while SFA-mediated toxicity and acute damage could not be excluded. P/O treatment didn’t show obvious cytotoxicity in both SIRT3OE and vector cells (Supplementary Figure S9). We speculated that SIRT3 activation improves mitochondrial function and enhances fatty acids oxidation against lipotoxicity. Meanwhile, SIRT3 reorganized the network of detyrosinated MTs to adopt a dispersed distribution, and to increase the close juxtaposition of LDs and mitochondria. The close juxtaposition could facilitate vesicle-free lipid transport into mitochondria directly, thereby preventing a systemic rise of cytosolic FFA levels and reducing lipotoxicity. In fact, the intimate contacts of LDs with mitochondria were reported in different cell types [66]. This juxtaposition could enable a direct, vesicle-free lipid transport between organelles [67]. Moreover, the increasing of kiss-and-run and relatively stable contacts between mitochondria and LDs were observed when detyrosinated MTs network reorganized via AMPK activation [39].

**Fig. 7:**
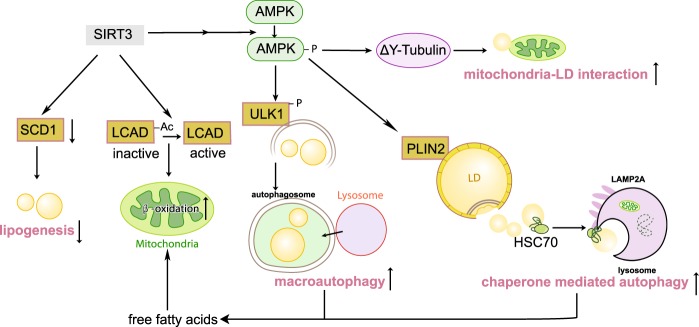
Schematic of the role of SIRT3 in protecting hepatocytes against lipotoxicity. SIRT3 activates AMPK to enhance macroautophagy and CMA on LDs and promote LDs dispersion on detyrosinated MTs, inhibits SCD1 expression to suppress lipogenesis, and deacetylates LCAD to increase fatty acid oxidation in mitochondria, resulting in attenuation of lipotoxicity in hepatocytes

Taken together, we discovered that the mitochondrial deacetylase SIRT3 enhances macroautophagy and CMA in LDs by activating AMPK, promotes LDs dispersion on detyrosinated MTs to increase mitochondrial energetics via AMPK activation, directly deacetylates LCAD to promote fatty acid β-oxidation, and inhibits SCD1 to suppress lipogenesis, resulting in amelioration of lipotoxicity in hepatocytes (Fig. 7). To the best of our knowledge it’s the first report providing the evidence that SIRT3 promotes macroautophagy and CMA to prevent hepatic lipid accumulation. SIRT3 might be a potential target in management of NAFLD.

## Supplementary information

Supplemental Information
