# Supplementary material for: SIRT3 promotes lipophagy and chaperon-mediated autophagy to protect hepatocytes against lipotoxicity
Source: Cell Death Differ. 2019 Jun 3;27(1):329–44. doi: 10.1038/s41418-019-0356-z (PMC7206074; doi:10.1038/s41418-019-0356-z)
Supplement: Supplementary file 1 — Supplemental Information [file 41418_2019_356_MOESM1_ESM.pdf]

Supplementary Information

SIRT3 promotes lipophagy and chaperon-mediated autophagy to protect hepatocytes against lipotoxicity

Tian Zhang, Jingxin Liu, Shengnan Shen, Qiang Tong, Xiaojun Ma and Ligen Lin

Supplementary Table 1 Antibodies used for Western blots.

Antibody	Source	Vendor	Catalog No.
**acetylated- lysine**	Mouse	Protein Technology	#9441
**AMPKα**	Rabbit	Cell Signaling Technology	#2532
**AMPKα1**	Rabbit	Cell Signaling Technology	#5832
**AMPKα2**	Rabbit	Cell Signaling Technology	#2757
**AMPKβ1**	Rabbit	Cell Signaling Technology	#12036
**AMPKβ2**	Rabbit	Cell Signaling Technology	#4148
***p*-AMPKα (Thr172)**	Rabbit	Cell Signaling Technology	#50081
**Atg5**	Rabbit	Cell signaling technology	#12994
**Beclin1**	Rabbit	Cell signaling technology	#3495
**detyrosinated α-tubulin**	Rabbit	Abcam	ab48389
**HSC70**	Rabbit	Protein Technology	10654-1-AP
**LAMP-2A**	Rabbit	Protein Technology	10397-1-AP
**LCAD**	Mouse	Cell Signaling Technology	#9796
**LC3**	Rabbit	Cell Signaling Technology	#12741
**PLIN2**	Rabbit	Protein Technology	15294-1-AP
**p62**	Rabbit	Protein Technology	18420-1-AP
**SCD1**	Rabbit	Cell signaling technology	#2794
**SIRT3**	Rabbit	Protein Technology	10099-1-AP
**ULK1**	Rabbit	Cell signaling technology	#8054
***p*-ULK1 (Ser555)**	Rabbit	Cell signaling technology	#5869
**α-tubulin**	Mouse	Santa Cruz Biotechnology	sc-8035
**β-actin**	Rabbit	Cell Signaling Technology	#8457

Supplementary Figure S1

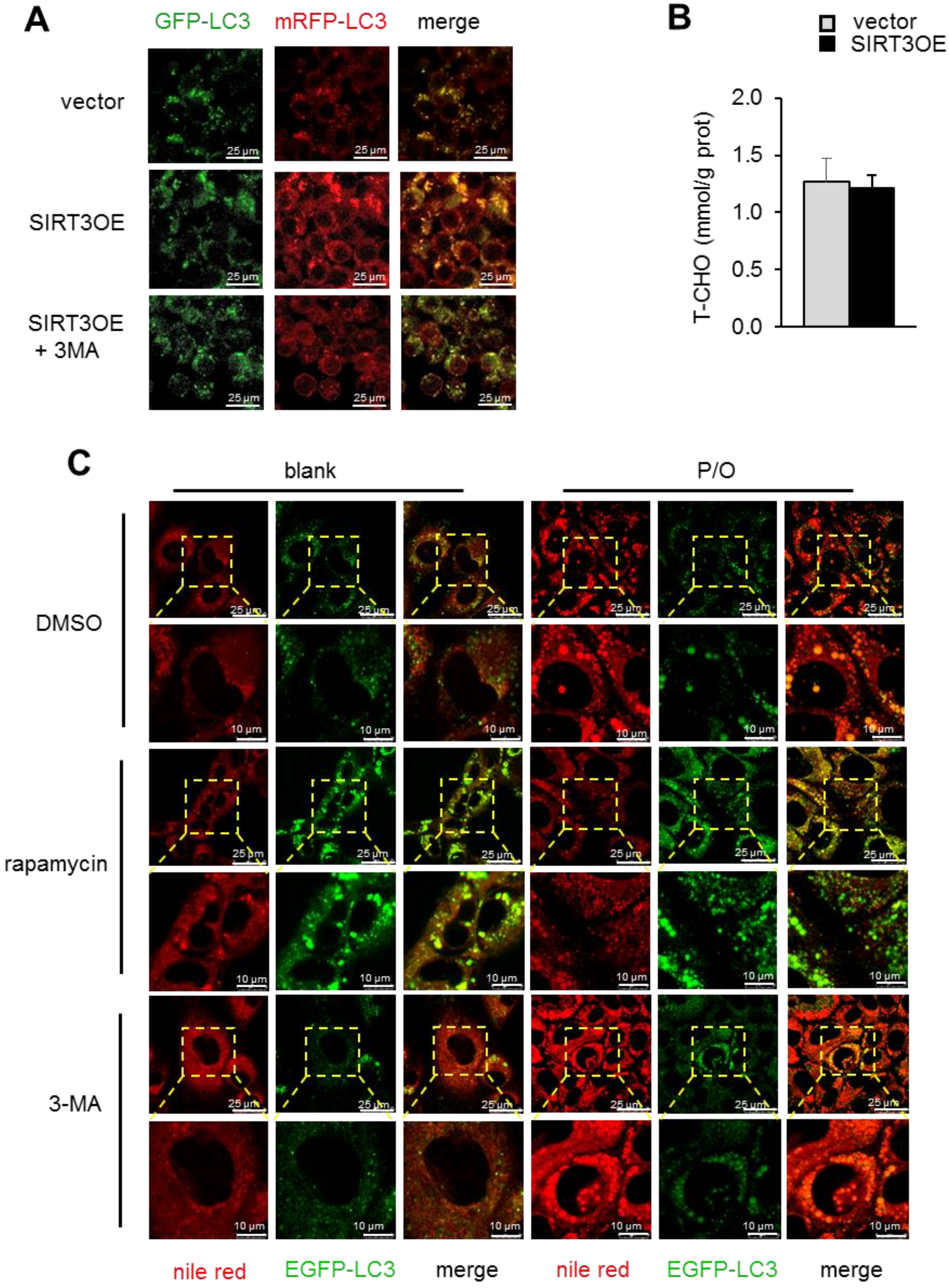

Supplementary Figure S1. a SIRT3OE and vector AML12 cells were infected with the Ad-

mCheryy-GFP-LC3. The mRFP-LC3 and GFP-LC3 puncta were examined by using a confocal microscope. Scale bar = 25 μm. **b** The cellular total cholesterol (T-CHO) level in SIRT3OE and vector hepatocytes treated with P/O mixture. **c** EGFP-LC3 puncta and LDs (Nile red) were observed in AML12 cells treated with DMSO, rapamycin or 3-MA. Scale bar =10 μm. Data are shown as mean ± S.D., *n* = 6.

Supplementary Figure S2

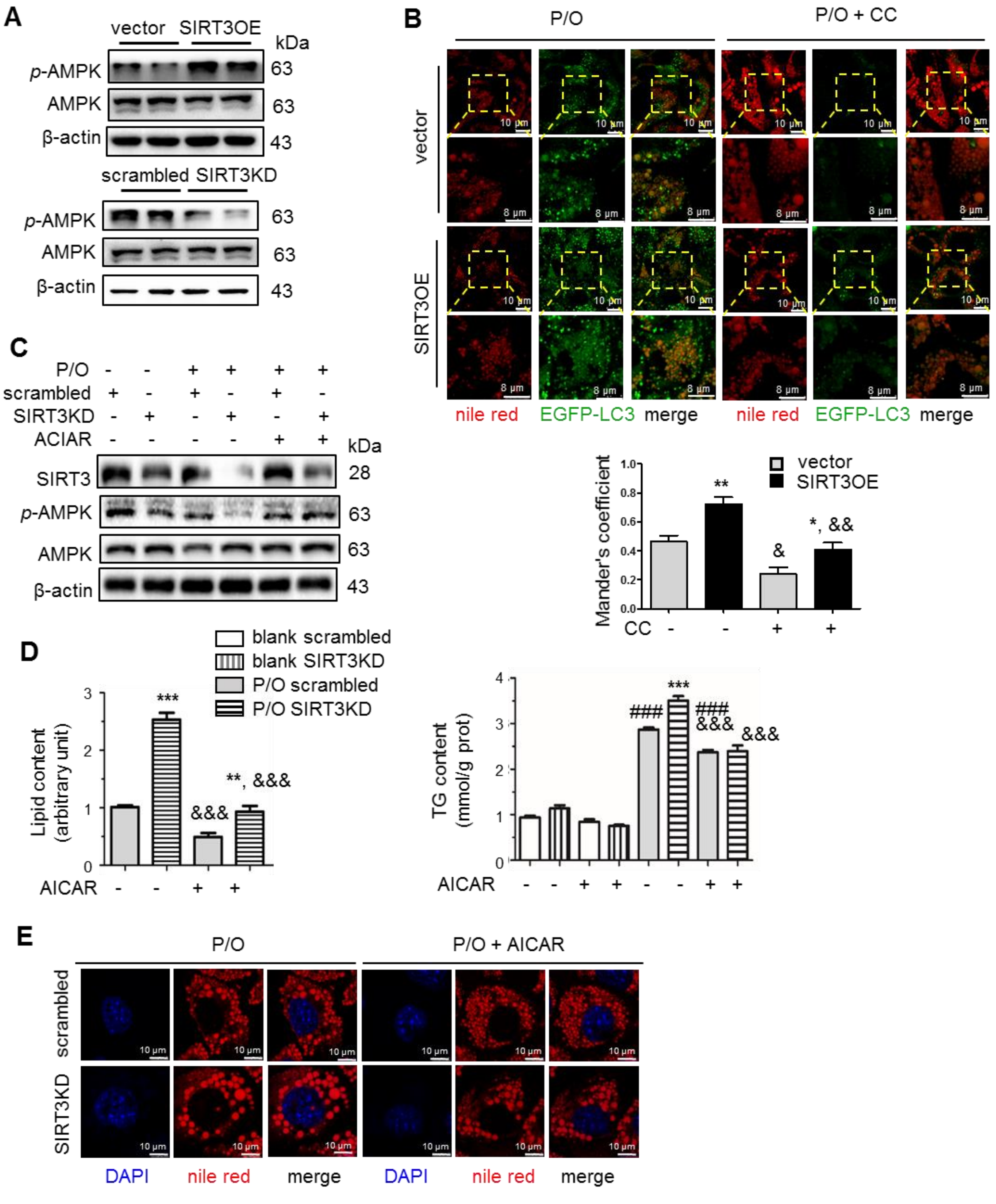

Supplementary Figure S2. a The phosphorylated AMPK and total AMPK levels in SIRT3OE and vector cells, as well as in SIRT3KD and scrambled cells. **b** EGFP-LC3 puncta and LDs (Nile red)

were observed in vector and SIRT3OE hepatocytes treated with and without CC. Scale bar = 10 μm. The Mander's overlapping coefficient of EGFP-LC3 and nile red. &*p* < 0.05, &&*p* < 0.01, DMSO vs. CC. **c** SIRT3, AMPK, and phosphorylated AMPK protein levels in scrambled and SIRT3KD hepatocytes treated with or without AICAR. **d** The lipid content and the cellular TG content in scrambled and SIRT3KD hepatocytes treated with or without AICAR. &&&*p* < 0.001, DMSO vs. AICAR. **e** Intracellular lipid content in scrambled and SIRT3KD hepatocytes treated with or without AICAR was visualized with nile red (red) staining. Nucleus were stained with DAPI (blue). Scale bar, 10 μm. Data are shown as mean ± S.D., $n = 6$, ***p* < 0.01 and ****p* < 0.001, scrambled vs. SIRT3KD. ###*p* < 0.001, blank vs. P/O treatment. One-way ANOVA was used to calculate the *p*-values.

Supplementary Figure S3

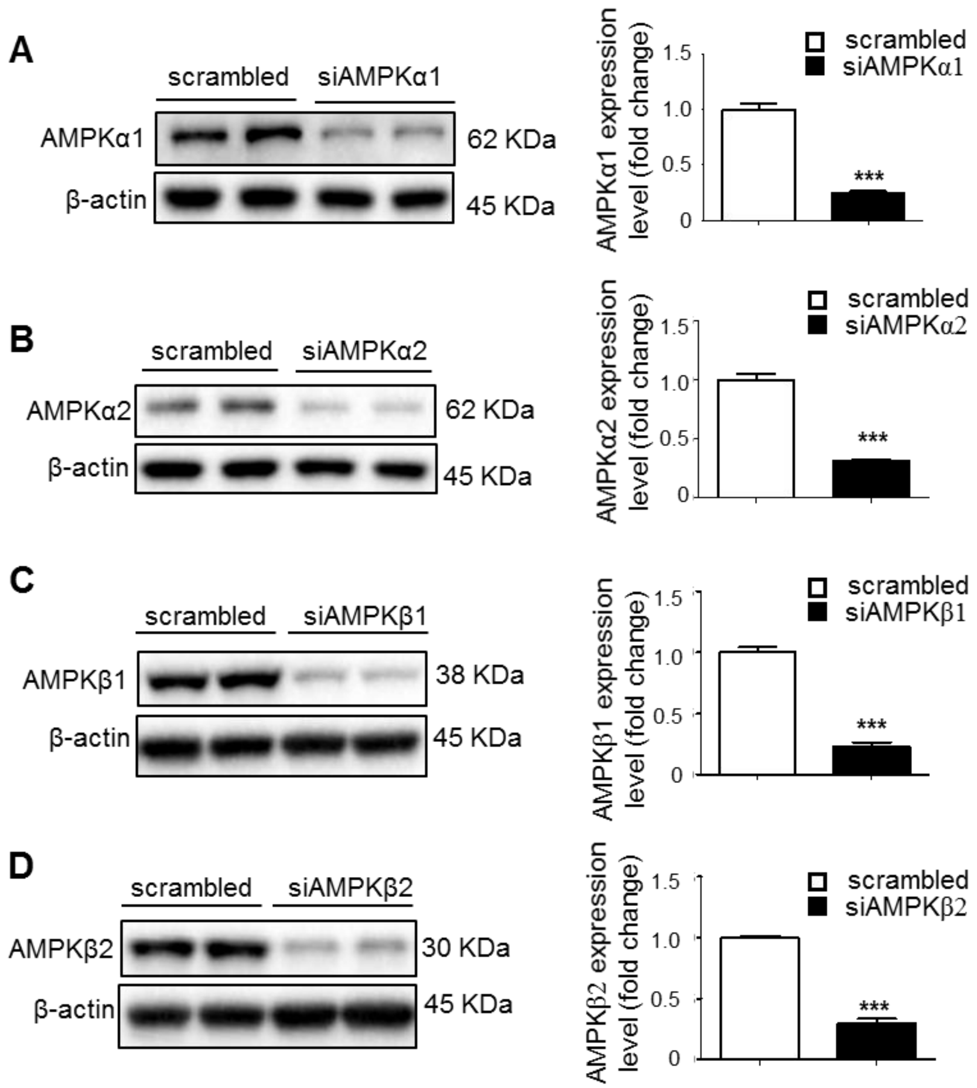

Supplementary Figure S3. a AMPKα1 expression in the scrambled and si*AMPKα1* cell lines. **b** AMPKα2 expression in the scrambled and si*AMPKα2* cell lines. **c** AMPKβ1 expression in the scrambled and si*AMPKβ1* cell lines. **d** AMPKβ2 expression in the scrambled and si*AMPKβ2* cell lines. Data are shown as mean ± S.D., $n = 5$, ***$p < 0.001$, scrambled vs. si*AMPKα1/α2/β1/β2*.

Supplementary Figure S4

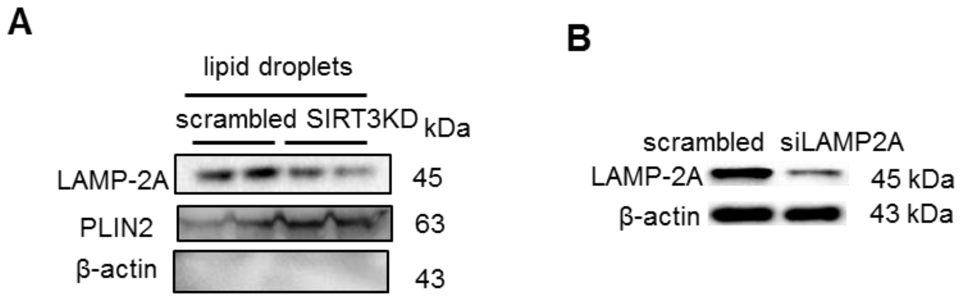

Supplementary Figure S4. a LAMP-2A and PLIN2 levels in LDs from SIRT3KD and scrambled cells treated with P/O mixture. **b** Generation of LAMP-2A knockdown cell line.

Supplementary Figure S5

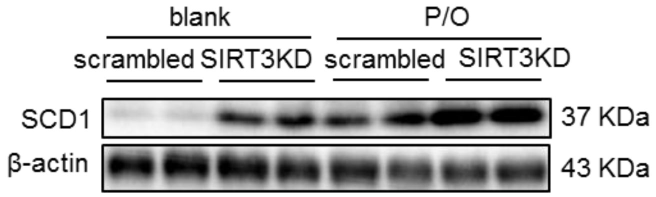

Supplementary Figure S5 SCD1 level in SIRT3KD and scrambled cells treated with or without P/O mixture.

Supplementary Figure S6

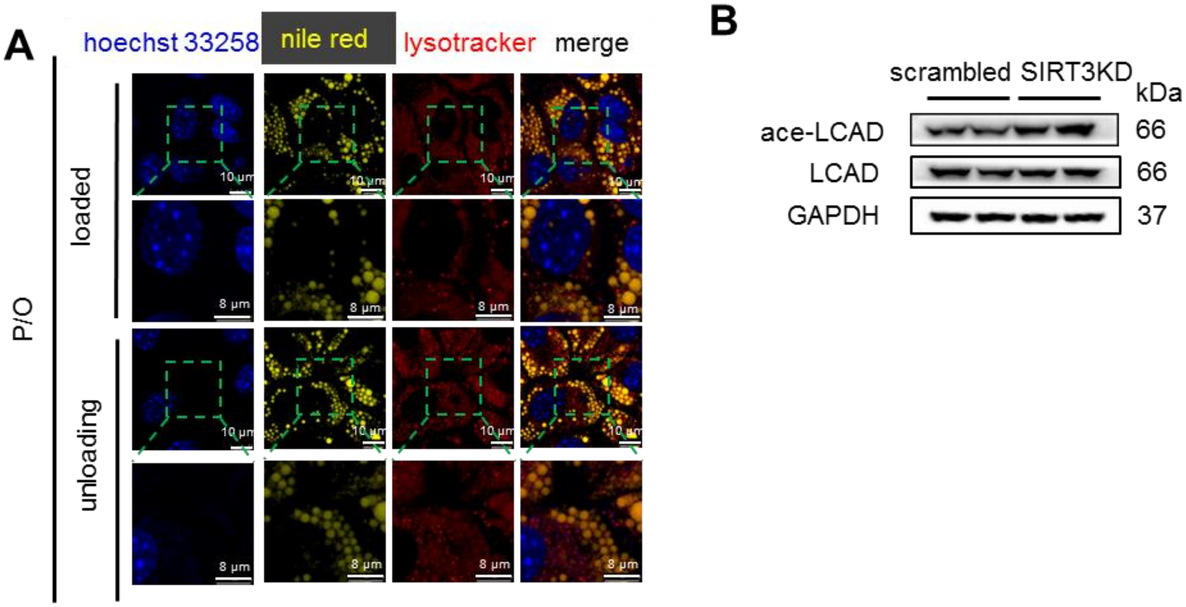

Supplementary Figure S6. a Confocal images of loaded and unloading cells. LDs were stained with Nile red (yellow), lysosomes were stained with lysotracker (red) and nucleus were stained with Hoechst 33258 (blue). Scale bar = 10 or 8 μm. **b** The acetylated and total LCAD levels in scrambled and SIRT3KD cells. Data are shown as mean ± S.D., $n = 6$.

Supplementary Figure S7

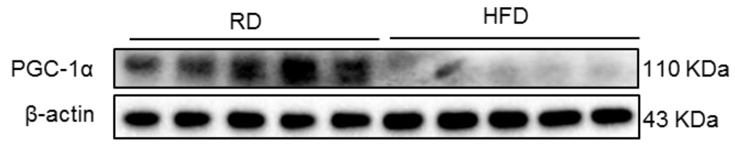

Supplementary Figure S7. PGC-1α protein level in primary hepatocytes from RD-fed and HFD-fed mice. The liver samples were collected from mice with the age of 26–28 weeks, fed with either RD or HFD for 18 weeks.

Supplementary Figure S8

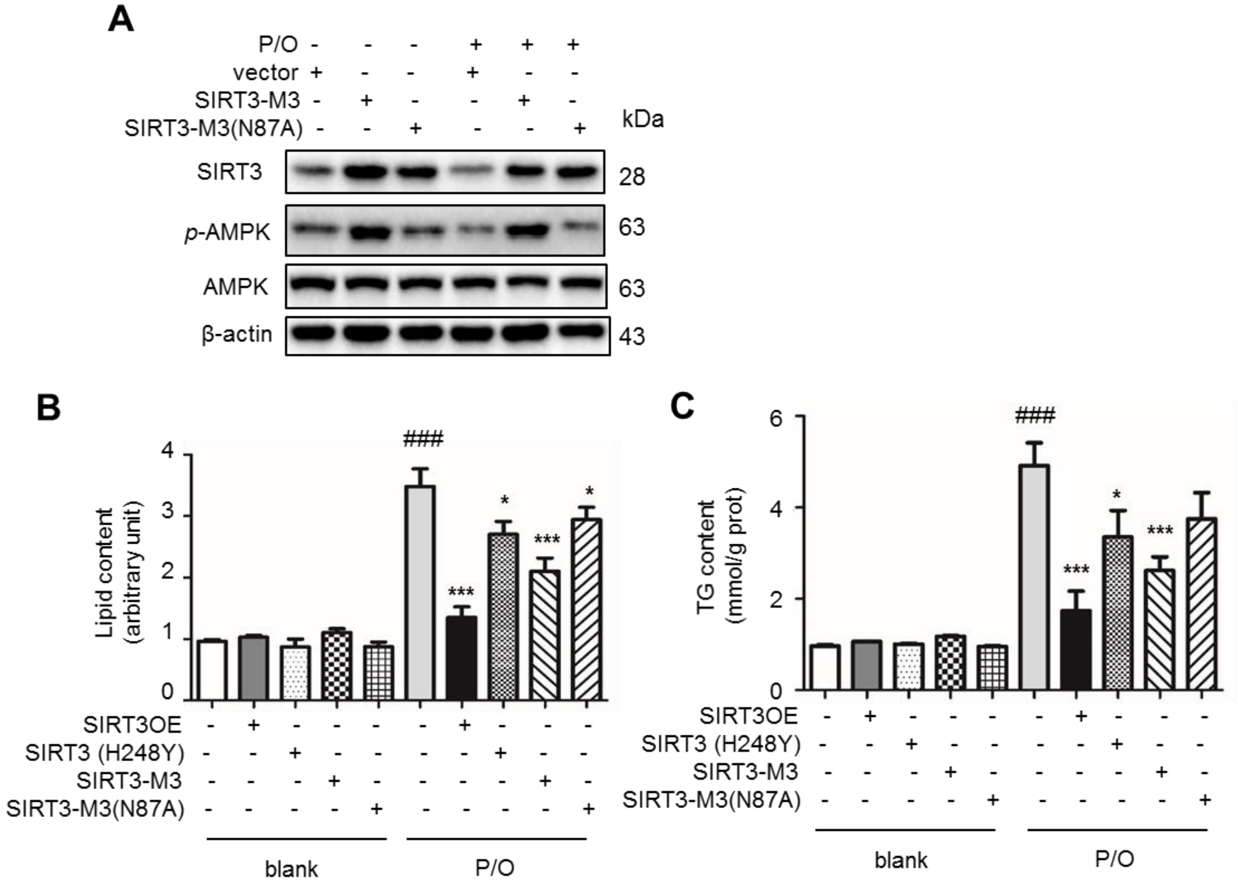

Supplementary Figure S8. a SIRT3, AMPK, and phosphorylated AMPK protein levels in vector, SIRT3-M3 overexpression and SIRT3-M3 (N87A) overexpression hepatocytes treated with or without P/O mixture. The lipid content (**b**) and the cellular TG content (**c**) in vector, SIRT3OE, SIRT3 (H248Y) overexpression, SIRT3-M3 overexpression and SIRT3-M3 (N87A) overexpression hepatocytes treated with or without P/O mixture. Data are shown as mean ± S.D., $n = 6$. *$p < 0.05$ and ***$p < 0.001$, vector vs. SIRT3OE, SIRT3 (H248Y) overexpression, SIRT3-M3 overexpression or SIRT3-M3 (N87A) overexpression. ###$p < 0.001$, blank vs. P/O treatment. One-way ANOVA was used to calculate the p-values.

Supplementary Figure S9

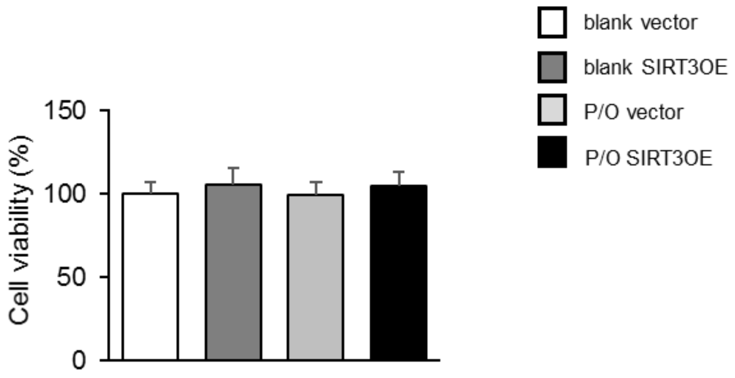

Supplementary Figure S9. The cell viability of SIRT3OE and vector cells treated with or without P/O mixture. Data are shown as mean ± S.D., *n* = 6.
